# Dissecting the mechanism of *NOP56* GGCCUG repeat-associated non-AUG translation using cell-free translation systems

**DOI:** 10.1016/j.jbc.2025.108360

**Published:** 2025-02-25

**Authors:** Mayuka Hasumi, Hayato Ito, Kodai Machida, Tatsuya Niwa, Tomoya Taminato, Yoshitaka Nagai, Hiroaki Imataka, Hideki Taguchi

**Affiliations:** 1 School of Life Science and Technology, Institute of Science Tokyo, Yokohama, Japan; 2 Department of Applied Chemistry, Graduate School of Engineering, University of Hyogo, Himeji, Hyogo, Japan; 3 Cell Biology Center, Institute of Integrated Research, Institute of Science Tokyo, Yokohama, Japan; 4 Department of Neurology, Kindai University Faculty of Medicine, Osaka-Sayama, Japan

**Keywords:** MyoD, O-GlcNAcylation modification, skeletal muscle, muscle fiber differentiation

## Abstract

The repeat expansion in the human genome contributes to neurodegenerative disorders such as spinocerebellar ataxia (SCA) and amyotrophic lateral sclerosis. Transcripts with repeat expansions undergo noncanonical translation called repeat-associated non-AUG (RAN) translation. The *NOP56* gene, implicated in SCA36, contains a GGCCTG repeat in its first intron. In tissues of patients with SCA36, poly (Gly-Pro) and poly (Pro-Arg) peptides, likely produced through *NOP56* RAN translation in (NOP56-RAN), have been detected. However, the detailed mechanism underlying NOP56-RAN remains unclear. To address this, we used cell-free translation systems to investigate the mechanism of NOP56-RAN and identified the following features. (i) Translation occurs in all reading frames of the sense strand of *NOP56* intron 1. (ii) Translation is initiated in a 5′ cap-dependent manner from near-cognate start codons upstream of the GGCCUG repeat in each frame. (iii) Longer GGCCUG repeats enhance NOP56-RAN. (iv) A frameshift occurs within the GGCCUG repeat. These findings provide insights into the similarities between NOP56-RAN and other types of RAN translation.

Spinocerebellar ataxia type36 (SCA36) is a neurodegenerative disease caused by a hexanucleotide repeat expansion (1, 2). Patients have been reported in specific regions, including Spain, Japan, and China (3, 4). The primary clinical features include hearing loss and tongue atrophy, along with motor neuron loss and muscle atrophy (1, 2, 5, 6). The GGCCTG hexanucleotide repeat expansion in intron 1 of the NOP56 gene, whose exon encodes a 60S ribosomal biogenesis protein, is associated with SCA36 (Fig. 1*A*). Healthy individuals have 5 to 14 GGCCTG repeats, whereas patients with SCA36 typically exhibit expansions ranging from 650 to 2500 repeats. Additionally, it has been reported that as few as 25 repeats can cause the disease (2, 5, 7). The GGCCUG expansion impairs splicing of the *NOP56* intron1, leading to the production of intron-retained transcripts (8). Indeed, transcripts with aberrant GGCCUG repeats have been detected in patient cells and are thought to be cytotoxic by forming RNA foci (1, 9, 10, 11, 12), a feature commonly observed in other repeat-associated neurodegenerative diseases (13, 14). In addition to this RNA-mediated toxicity, a noncanonical process known as repeat-associated non-AUG (RAN) translation contributes to SCA36 pathogenesis (8, 10).

**Figure 1:**
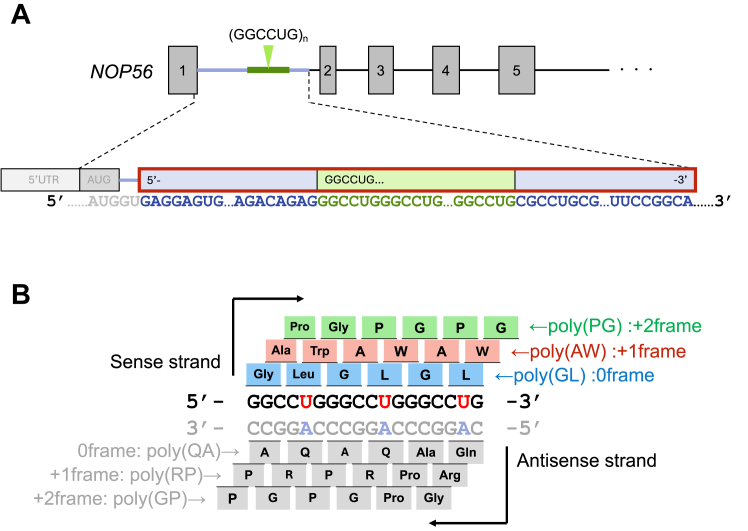
***NOP56* gene structure and the GGCCUG-derived putative dipeptide repeats**. *A*, *NOP56* gene structure. The GGCCUG repeat in intron 1 of the *NOP56 gene* and its flanking regions are shown in detail. The red box highlights the region used in this study. *B*, schematic of putative dipeptide repeats derived from the *NOP56* GGCCUG repeat.

RAN translation, first identified in spinocerebellar ataxia type 8 (SCA8) and myotonic dystrophy type 1 (DM1) (15), is implicated in various neurodegenerative diseases associated with repeat expansions (14, 16). While the molecular mechanisms of RAN translation vary among diseases, a common feature is the involvement of mRNA secondary structures, such as G-quadruplex (G4) and hairpin structures, in the GC-rich repeat expansion region (17). The initiation of C9-RAN (*C9orf72*, GGGGCC) and CGG-RAN (*FMR1*, CGG) is 5′-cap dependent (18, 19), starting from near-cognate AUG codons upstream of the repeat sequences in each reading frame, facilitated by scanning mechanisms involving eIF4A and eIF4F (18, 19, 20, 21, 22, 23). Similarly, SCA3-RAN (*ATXN3*, CAG) initiates from non-cognate codons (*e*.*g*., CUU, ACU) upstream of the repeat sequences (24). In addition to cap-dependent translation, C9-RAN also employs a cap-independent mechanism resembling an internal ribosomal entry site (IRES), as shown using bicistronic reporter assays (25, 26). Ribosomal frameshifting during elongation on repeat sequences has also been observed, such as GA-to-GP frameshifts in C9-RAN (19, 27, 28) and R-to-G frameshifts in CGG-RAN (29)). These frameshifts result in chimeric peptides with distinct subcellular localization and toxicity compared to non-frameshift peptides (8, 29). Furthermore, the integrated stress response (ISR) enhances the translation efficiency of C9-RAN, CGG-RAN, and SCA3-RAN (20, 24).

Regarding SCA36, the GGCCTG repeat in the *NOP56* gene has been identified in patient cells and mice (8). Possible RAN translation products of this repeat include poly Gly-Leu (GL, from GGC-CUG, 0 frame), poly Ala-Trp (AW, from GCC-UGG, +1 frame), Pro-Gly (PG from CCU-GGG, +2 frame) dipeptide repeats (DPRs) on the sense strand and poly Arg-Pro (RP), poly Glu-Ala (QA), and poly Pro-Gly (GP) DPRs on the antisense strand (Fig. 1*B*). Poly GP and poly PR products translated in *NOP56* GGCCUG repeat sequence have been detected in cells of patients with SCA36 (10). McEachin *et al*. (8) reported that the AUG start codon in exon1 of *NOP56* predominantly generates poly PG DPR. In the absence of the exon1 AUG start codon, poly GL, poly AW, and poly GP are also translated from the GGCCUG repeat (10). However, the detailed mechanisms of non-AUG translation initiation, including the specific initiation sites, remain unclear.

Here, we investigated the initiation and elongation mechanisms of the *NOP56*-GGCCUG RAN translation (NOP56-RAN) using the repeat without an AUG start codon in several cell-free translation systems, based on our recent study of C9-RAN (27). Reporter assays with Western blotting and nano luciferase (Nluc) revealed several features of NOP56-RAN, including the identification of initiation sites, 5′ cap-dependency, and the influence of longer repeats. These findings provide new insights into RAN translation, potentially contributing to therapeutic strategies for SCA36.

## Results

### NOP56-RAN reporter series using nano-luciferase

**Figure 2:**
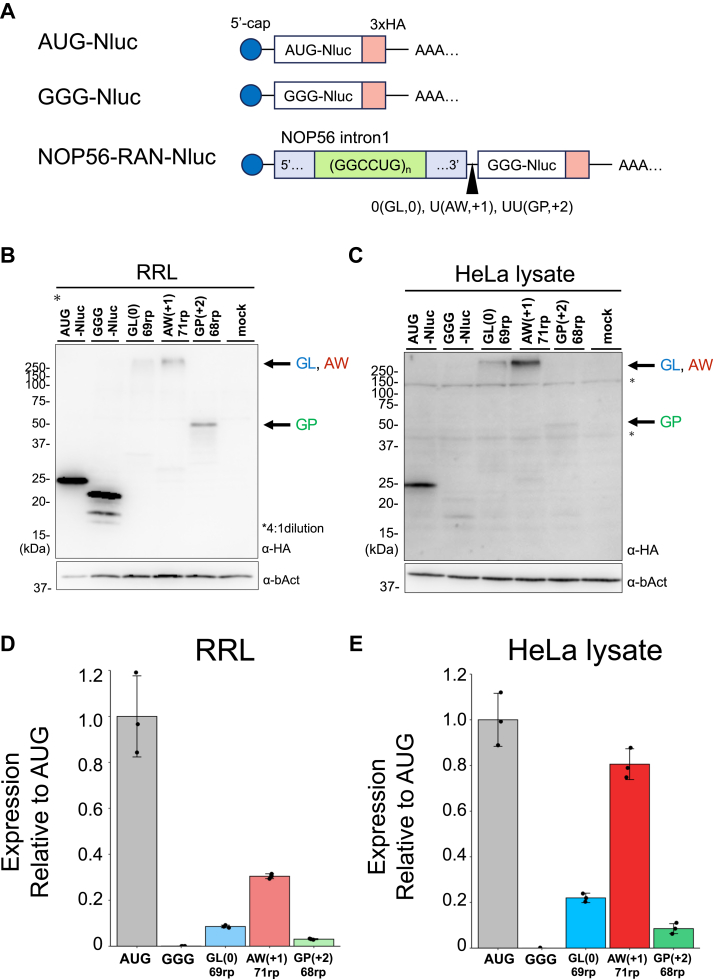
**NOP56-RAN translation in cell-free translation systems using nano-luciferase reporter**. *A*, schematic of nano-luciferase (Nluc)-3 × HA reporters. Nluc was used as a reporter enzyme to quantify translation efficiency. *B* and *C*, anti-HA Western blot of translation products in each cell-free translation system using the Nluc reporters. *B*, Rabbit reticulocyte lysate (RRL), (*C*) HeLa lysate. Asterisks (∗) indicate non-specific bands. Predicted molecular sizes: AUG-Nluc: 25 kDa, GL (0): 41 kDa, AW (+1): 46 kDa, and GP (+2): 39 kDa. Anti-β-actin (bAct) was used as a loading control. *D* and *E*, expression of NOP56-Nluc reporters normalized to GGG-Nluc expressed in cell-free translation systems. *D*, RRL and (*E*) HeLa lysate. Error bars represent standard deviations (±SD) from three independent experiments.

To investigate the detailed mechanism of NOP56-RAN, we developed a reporter series using only intron1 of the *NOP56* gene, which lacks an AUG codon in any reading frame (Figs 1*A*, 2*A*). This reporter system fused nano-luciferase (Nluc) and a tandem-HA tag downstream of the GGCCUG repeat. To examine all three possible reading frames, we inserted one or two nucleotides between the repeat and Nluc (0 nt: GL (0), +1 nt: AW (+1), +2 nt: GP (+2)) (Fig. 2*A*).

Translation of the reporter series containing 69 to 71 GGCCUG repeats, we performed *in vitro* translation in HeLa lysate and rabbit reticulocyte lysate (RRL) with mRNA containing a 5′-cap structure. Western blotting detected translation products from all three frames, although the GP frame products were faint in HeLa lysate (Fig. 2, *B* and *C*). Consistent with previous reports (8, 10), products from the GL (0) and AW (+1) frames exhibited higher molecular weights than their predicted sizes (∼40 kDa for GL, ∼48 kDa for AW) (Fig. 2, *B* and *C*). The presence of these larger products became more pronounced as the repeat length increased (Fig. S1*A*, *B*), suggesting that longer GL and AW DPRs are aggregation-prone, likely due to their higher hydrophobicity. Notably, overall translation efficiency was higher in RRL than in HeLa lysate (Fig. S1*C*).

For the luciferase activity of this reporter system, all frames showed 100- to 1000-fold higher activity compared to the control reporter containing GGG-Nluc (Fig. 2, *D* and *E*). The highest translation activity was observed in the AW frame, reaching approximately 30% (RRL) or 80% (HeLa lysate) of the activity of AUG + Nluc, a positive control, indicating robust translation even without a canonical AUG start codon. Moreover, we inserted a self-cleaving T2A peptide (30) between DPR and Nluc to investigate whether the aggregation of AW and GL affects Nluc activity (Fig. S2*A*). As a result, the self-cleavage of DPR enhanced by 1.5-fold Nluc activity in both AW and GL (Fig. S2, *B–C*). However, this enhancement was insufficient to account for the differences in translation efficiency between AW, GL, and GP (Fig. 2, *D* and *E*).

### NOP56-RAN utilizes upstream AUG-like codons as initiation sites in each frame

We next examined the 5′ m^7^GpppG-cap dependency in NOP56-RAN and found that translation with a 5′ ApppG-cap, a cap analog, in HeLa lysate almost abolished (Fig. S3*A*). This 5′-cap dependency was further confirmed by inhibited translation in the presence of free m^7^GpppG compound (Fig. S3*B*), which competitively inhibits eIF4E binding.

**Figure 3:**
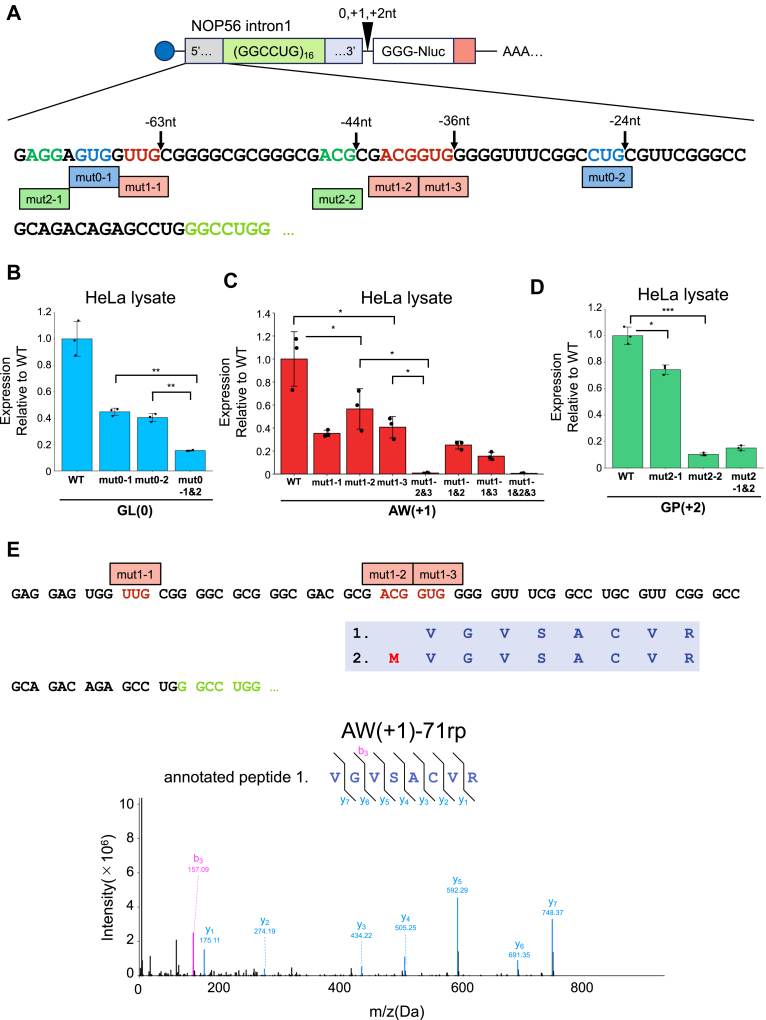
**Identification of initiation sites in each reading frame**. *A*, mutations to identify NOP56-RAN start codons. The GGCCUG repeat length is 16 repeats. For example, “*mut0-1*” refers to the first candidate start codon in the 0 frame. (*B*-*D*) Expression of the reporters with mutation relative to the wild-type (WT) reporter expressed in HeLa lysate. *B*, GL (0), (*C*) AW (+1) and (*D*) GP (+2). Error bars represent ±SD from three independent experiments. ∗*p* < 0.05; ∗∗*p* < 0.01; ∗∗∗*p* < 0.001, two tailed Student’s *t* test. *E*, LC-MS/MS analysis of the N-terminal region of the HA-immunoprecipitated and trypsin-digested poly AW-71 repeat protein expressed in RRL. LC-MS/MS analysis identified two peptides, 1-VGVSACVR, 2-MVGVSACVR. The MS spectrum for peptide 1 is shown, with related MS spectra provided in Fig. S5.

Based on the 5′-cap dependency, NOP56-RAN is likely initiated from specific sites upstream of the GGCCUG repeat, similar to findings in SCA3-RAN experiments (24). Translation of a series of reporters with various truncations of the 5′ end of *NOP56* intron1 showed an overall decrease in translation efficiency (Fig. S3, *C–F*). Based on these results, we generated reporter mRNAs with mutations at the predicted near-cognate start codons, replacing them with AAA (non-initiation) codons, and tested them in conjugation with (GGCCUG)_16_ repeats (Fig. 3*A*). The results for each frame were as follows.

i) GL (0)-frame: In the GL (0)-frame, mutations in either GUG (mut 0–1) or CUG (mut 0–2) reduced luciferase activity by approximately 60%, and by ∼80% when both mutations were introduced (HeLa lysate; Fig. 3*B*, RRL; Fig. S4*A*), suggesting that either GUG or CUG serves as the initiation codon. Additionally, Western blotting detected different molecular sizes of translation products in each mutant (Fig. S4*D*), supporting the use of two initiation sites.
ii) AW (+1)-frame: All three possible mutations in the near-cognate start codons decreased luciferase activity by 40 to 60% (HeLa lysate; Figs. 3*C*, RRL; S4*B*, *E*). Combining mutations in the two near-cognate start codons showed that the double mutation of ACG (mut 1–2) and GUG (mut 1–3) nearly eliminated luciferase activity (Figs. 3*C*, S4*B*, *E*), suggesting that ACG and GUG are critical for AW (+1)-frame initiation.
  To obtain further evidence, we performed LC-MS/MS analysis of the AW (+1)-frame initiation site, as previously applied in other RAN translation studies (21, 22, 24). We translated NOP56-RAN reporters with 16 or 71 GGCCUG repeats in RRL, immunoprecipitated the translation products with HA beads, digested them with a trypsin/LysC mix, and analyzed the peptides by LC-MS/MS. We identified two unique peptides from the N-terminal region of the NOP56-RAN AW (+1)-frame, VGVSACVR and MVGVSACVR (Fig. 3*E*, Fig. S5, *A–C*). These peptides were predicted to initiate from the ACG codon, with and without the removal of the N-terminal methionine, strongly suggesting that the AW (+1)-frame is primarily initiated from the ACG codon (mut 1–2).

iii) GP (+2)-frame: Of the two possible near-cognate start codons, mutation in ACG (mut 2–2) significantly decreased luciferase activity (HeLa lysate; Figs. 3*D*, RRL; S4, *C*, *F*), indicating that the +2 frame initiates from the ACG codon.

Taken together, NOP56-RAN uses specific near-cognate codons upstream of the GGCCUG repeat in each reading frame as initiation sites.

Next, we investigated whether putative initiation codons affect the translation of other frames in HeLa lysate. Mutating the putative initiation codons for the GL (0)-frames enhanced the translation efficiency of the AW (+1)-frame (Fig. S6), suggesting that translation in the AW (+1)-frame is suppressed by translation in the GL (0)-frame.

### NOP56-RAN is enhanced by longer GGCCUG repeats

**Figure 4:**
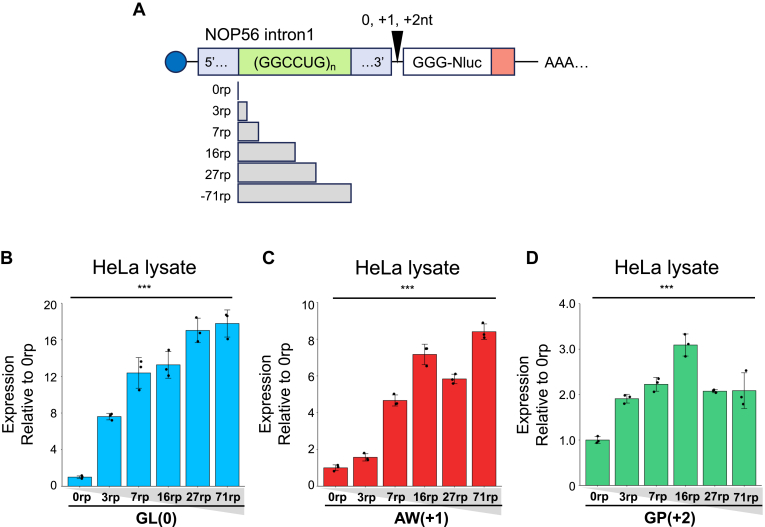
**Repeat length dependency in NOP56-RAN**. *A*, schematic of the NOP56-RAN reporter with varying repeat (rp) numbers in each frame. *B-D*, relative expression levels of NOP56-RAN reporters normalized to the 0 repeat in HeLa lysate. *B*, GL (0), (*C*) AW (+1), and (D) GP (+2). Error bars represent ±SD from three independent experiments. ∗∗∗*p* < 0.001, one-way ANOVA with Dunnett’s multiple comparison test.

Studies on RAN translation have shown that longer RNA repeat expansions, such as the GGGGCC repeats in *C9orf72*, enhance translation efficiency (15, 18, 27, 31, 32, 33). To investigate this repeat length dependency in NOP56-RAN, we created a reporter series with different GGCCUG repeat lengths (0–71 repeats) (Fig. 4*A*). In HeLa lysate, translation in all frames was more efficient in the mRNA with 3 repeats than with no GGCCUG repeat (=0 repeat) (Fig. 4, *B–D*). Notably, in the GL (0) frame, the translation efficiency with 3 repeats was more than 5-fold higher (Fig. 4*B*). Further repeat expansion generally increased translation efficiency in all frames, although no increase was observed in the GP (+2) frame with more than 16 repeats (Fig. 4, *B–D*).

### The AW (+1)-to-GP (+2) frameshift occurs within *NOP56* intron1

**Figure 5:**
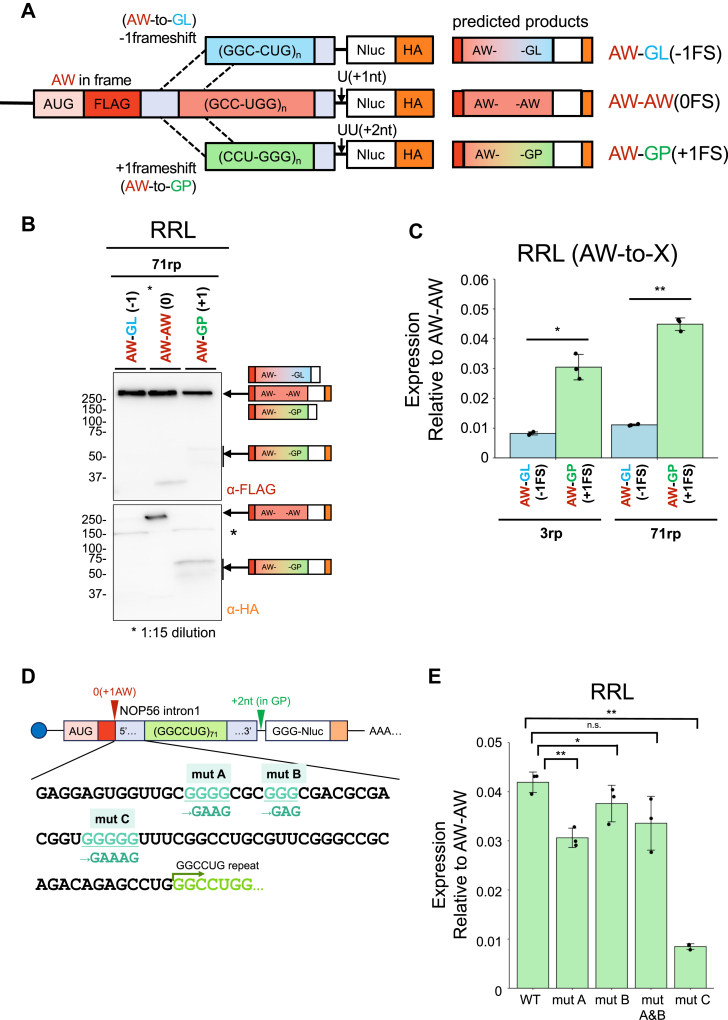
**Frameshift analysis in NOP56-RAN**. *A,* Schematic of reporters with an AUG driving expression through an N-terminal FLAG (red) tag in AW (+1), followed by a C-terminal 3 × HA (orange) tag fused to the NOP56 intron 1 with 3 or 71 repeats (rp). *B*, Anti-FLAG and HA Western blot of the FLAG (AW)-NOP56-Nluc, HA reporter mRNAs expressed in each frame in RRL. Asterisks (∗) indicate non-specific bands. Predicted molecular sizes for anti-FLAG: AW-GL and AW-GP (+2) are smaller than 46 kDa (71 rp); AW-AW in frame: 46 kDa or high molecular weight (71 rp). Predicted molecular sizes for anti-HA: AW-GL not detected; AW-AW in frame: 46 kDa; AW-GP: 40 to 47 kDa. *C*, Luciferase activity of frameshifted products (AW-to-GL, AW-to-GP) expressed in RRL. Relative values are shown when the luciferase activity for in-frame (AW-to-AW) is set to 1. Error bars represent ±SD from three independent experiments. ∗*p* < 0.05; ∗∗*p* < 0.01, two tailed Student’s *t* test. *D*, Schematic of reporters with mutations at putative frameshift sites. *E*, Frameshift efficiency of the AW-to-GP mutations at putative frameshift sites. Relative expression of AW-AW expressed in RRL. Error bars represent ±SD from three independent experiments. n.s. = not significant; ∗*p* < 0.05; ∗∗*p* < 0.01, two tailed Student’s *t* test.

Next, we investigated the potential for a frameshift in NOP56-RAN. We focused on a frameshift from the AW (+1)-frame to other frames, as translation from the +1 frame was most efficient in our assay system. We engineered a dual-tagged reporter series, in which the AUG start codon was inserted in-frame in the AW (+1)-frame, upstream of the *NOP56* intron1 (Fig. 5*A*). For detection, we introduced a FLAG tag in the N-terminal region and Nluc-HA downstream of the repeat sequence, adding one or two nucleotides to distinguish frameshift products (Fig. 5*A*).

Western blotting after translation using RRL showed that the construct for the AW-to-GP frameshift (+1 to +2) produced faint ∼50 kDa protein bands in both FLAG and HA detection (Fig. 5*B*). The ∼50 kDa protein corresponds to the approximate calculated size (∼40 kDa) for the AW-to-GP frameshifted product. Additionally, no large band (>250 kDa) was observed in the anti-HA detection for the GP (+2) construct, which would indicate a non-frameshifted AW-frame product, as seen in Figure 2. These results suggest an AW-to-GP frameshift under the tested conditions. The luciferase activities for the AW-to-GP and AW-to-GL frameshift constructs relative to the AW-to-AW construct in the 71 repeat constructs were 4.0% and 1.1%, respectively (Fig. 5*C*), indicating that the AW-to-GP frameshift predominates. The frameshift efficiencies in the 71 repeat series were higher than those in the 3-repeat series (AW-to-GP: 2.5%, AW-to-GL: 0.8%) (Fig. 5*C*). In addition to the AW-to-GP frameshift, we also investigated the possibility of a GL-to-GP frameshift. The luciferase reporter assay for X-to-GP frameshifts in 71 repeats were 1.2% and 4.0%, respectively. These frameshift efficiencies in 71 repeats were higher than those in 3 repeats (GL-to-GP: 0.6%, AW-to-GP: 2.5%) (Fig. S7, *A–C*). Although these analyses were performed using RRL due to the large amount of translation products required for the immunoblotting, the luciferase assay showed similar trends when using HeLa lysate (Fig. S7*D*, *E*).

Furthermore, we sought to identify the site of the AW-to-GP frameshift. It is known that G or C runs can induce +1 frameshift (19, 29, 34, 35, 36), and both C9-RAN and CGG-RAN are associated with +1 frameshifts (19, 29, 34, 35, 36). Therefore, we hypothesized that the frameshift might occur at consecutive G or C sequences upstream of the repeat in NOP56-RAN. We identified several G-runs (GGGG, GGG, and GGGGG, Fig. 5*D*) upstream of the repeat region and mutated them to GAAG (mut A), GAG (mut B), and GAAAG (mut C), respectively (Fig. 5*D*). When these reporter RNAs were translated in RRL, the frameshift efficiency decreased from 4% to 1% when the GGGGG sequence was mutated (mut C) (Fig. 5*E*), as confirmed by Western blotting (Fig. S8*A*). Similarly, the frameshift efficiency decreased from 2% to 1% in HeLa lysate (Fig. S8*B*). These results suggest that the G-rich region upstream of the repeat contributes to the AW-to-GP frameshift.

### NOP56-RAN in human cultured cells

**Figure 6:**
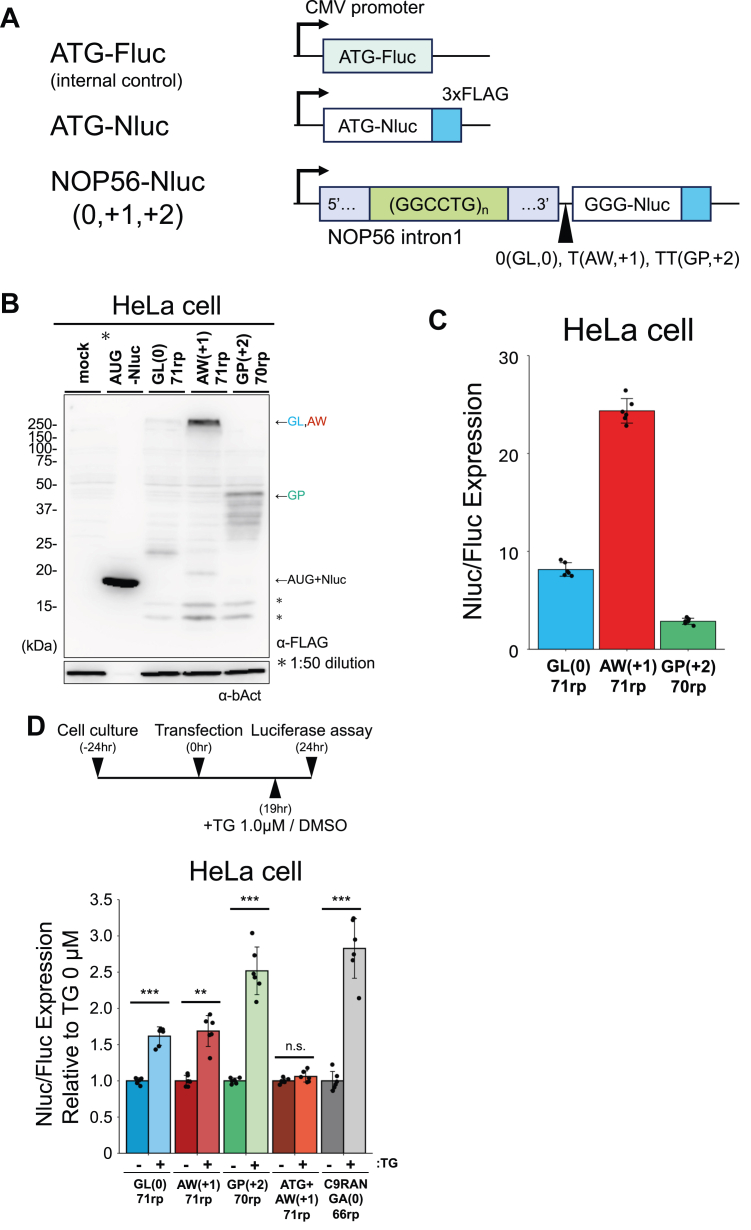
**NOP56-RAN in human cultured cells**. *A*, schematic of Nluc-3 × FLAG reporters. Nluc was used as a reporter enzyme to quantify translation efficiency. ATG-Fluc was included in all assays for normalization. *B*, anti-FLAG Western blot of the NOP56-Nluc reporter expressed in HeLa cells. Asterisks (∗) indicate non-specific bands. Predicted molecular sizes of the products: AUG + Nluc: 21 kDa, GL (0): 38 kDa, AW (+1): 43 kDa, and GP (+2): 36 kDa. *C*, relative expression of the NOP56-Nluc reporters normalized to ATG-Fluc in HeLa cells. Error bars represent standard deviations (±SD) from six independent experiments. *D*, *top*, schematic of stress induction by thapsigargin (TG). *Bottom*, relative Nluc/Fluc expression of the NOP56-Nluc reporters normalized to the HeLa cells without TG. Error bars represent standard deviations (±SD) from six independent experiments. n.s. = not significant; ∗∗*p* < 0.01; ∗∗∗*p* < 0.001, two tailed Student’s *t* test.

Finally, we investigated the translation of the GGCCUG repeat in HeLa cells to compare the results obtained by cell-free systems with those in cells. The Nluc reporters, including *NOP56* intron1 with 71 GGCCUG repeats, were transfected into HeLa cells along with a plasmid carrying firefly luciferase (Fluc) for normalization (Fig. 6*A*). Western blotting revealed translation products in all frames; both AW and GL, though in low amounts, formed high molecular weight oligomers (Fig. 6*B*), similar to the cell-free translation conditions. The dual-luciferase reporter assay revealed that the AW (+1) frame was the most prominently translated, with ∼3 and 10-fold higher translation than the GL (0) and GP (+2) frames, respectively (Figs. 6*C*, S9*A*). The trend observed in HeLa cell translation was consistent with the cell-free translation results (Fig. 2*E*).

We also tested whether ISR stimulates NOP56-RAN translation, as observed in other RAN translations (20, 24, 25). Remarkably, the addition of thapsigargin (TG), which induces ER stress, increased translation of all frames by 1.6 to 2.5-fold (Fig. 6*D*). This stimulation is likely due to ISR-mediated activation of non-AUG start codons, as TG enhanced the translation of near-cognate start codons, ACG and CUG (Fig. S9*C*).

## Discussion

Here, we investigated the cell-free translation of *NOP56* GGCCUG repeats by developing a NOP56-RAN reporter series from sequences surrounding the GGCCUG in the *NOP56* intron1. Although previous studies have reported that all frames can be translated from the GGCCUG repeats (8, 10), we examined the detailed mechanism of NOP56-RAN in the same reporter system using constructs lacking AUG upstream of intron1.

Using this reporter system, we identified putative initiation codons for NOP56-RAN in three frames: CUG and GUG in the GL (0)-frame, primarily ACG in the AW (+1)-frame, and GP (+2)-frame. In the AW (+1)-frame, MS analysis identified two peptides, VGVSACVR and MVGVSACVR, in the potential N-terminal regions, strongly suggesting that the methionine is incorporated at ACG as the start codon. Similar to other RAN translations, CUG in the GA frame and AGG in the GR frame in C9-RAN, ACG and GUG in the G frame in CGG-RAN, and CUU and ACU in the Q frame in SCA3-RAN have been identified as start codons (21, 22, 24). Taken together, these results suggest that NOP56-RAN utilizes multiple potential initiation sites in all frames upstream of the GGCCUG repeats.

By translating the three frames under the same conditions, the AW (+1)-frame was found to be the most efficient. This may be due to the presence of multiple non-AUG codons in the region before the GGCCUG repeat sequence. In the AW (+1) frame, ACG in (GCG)ACG(G) was identified as the start codon, and the sequence flanking ACG closely resembles a typical Kozak sequence (GCCAUGG) (37). Moreover, it is known that the distance from the initiation codon with a Kozak sequence to bulky secondary structures, such as repeat sequences, is critical for initiating translation (38, 39). In NOP56-RAN, the near-cognate start codon ACG, located closest to the bulky GGCCUG repeat (−36 nt), aligns with the Kozak consensus sequence. This likely accounts for the higher translation efficiency of the AW (+1) frame.

Non-AUG translation, including RAN translation, is known to be enhanced by ISR (20, 25, 40), as observed in NOP56-RAN. One reason for this enhancement is the phosphorylation of eIF2α, a translation initiation factor (41, 42, 43). ISR-induced phosphorylation of eIF2α is thought to suppress global canonical AUG-start translation and promotes noncanonical translation, such as non-AUG initiation (44, 45). In addition to eIF2α, ISR also modifies other translation factors, including eIF2B (46, 47) and eIF3d (48). Although the impact of these factors on RAN translation remains unclear, it is possible that the global remodeling of translation landscape by ISR may enhance non-AUG translation by altering the relative abundance of translation-related factors.

As with other RAN translations, NOP56-RAN is overall facilitated by longer repeats. This repeat-length dependence may be due to the bulky secondary structure formed by the repeat RNA (39, 49). Indeed, the GGCCUG repeat in the *NOP56* gene is thought to form a G4 structure (9, 12), similar to the GGGGCC repeat in C9-RAN (19, 26, 27).

We observed a +1 frameshift from the AW (+1) to the GP (+2) frame in NOP56-RAN. In general, the mechanisms of −1 and +1frameshifts differ. The −1 frameshift is primarily involved in viral replication control and occurs in U-rich regions (slippery sequences) just before sequences that form pseudoknot structures (50, 51, 52, 53). In contrast, the +1 frameshift, including GA-to-GP and GR-to-GA in C9-RAN, R-to-G in CGG-RAN (19, 27, 29), is thought to be induced by ribosome stalling within repeat RNAs with certain structures, such as G4 (34, 54). The ability of GGCCUG repeats to form G4 RNA structures may contribute to the +1 frameshift.

We observed repeat-length stimulation of the AW-to-GP frameshift, part of which was not abolished by an upstream mutation (Fig. 5, *C* and *E*), suggesting that the frameshift occurs within the repeat, as seen in C9-RAN (19, 28). Based on this and previous reports of frameshifts in RAN translation, it can be predicted that frameshifts occur within repeats in guanine-rich regions, albeit as minor events (∼4%).

Although this study was primarily conducted in a cell-free system, it provides valuable insights into the mechanism of NOP56-RAN. While the cell-free translation system used in this study employed extract-based systems, we have recently developed a system to investigate RAN translation (C9-RAN) using a reconstituted translation system with only human-derived translation factors (Human PURE system (55, 56)). Using the Human PURE system as an extension of this study, a more detailed mechanism could be explored. For example, reproducing the changes in the proportion of initiating factors, such as eIF2B, and eIF3d, during ISR may help identify potential therapeutic targets. Finally, analyzing the detailed molecular mechanisms of different RAN translations will highlight both similarities and differences, which may help uncover new eukaryotic translation mechanisms unrelated to disease.

## Materials and methods

### Plasmids

All plasmids were constructed using standard cloning procedures and Gibson assembly. To maintain the repeat size, we used SURE2 *Escherichia coli* strain for all plasmid preparations. Detailed information on plasmids and primers is summarized in Tables S1 and S2. Briefly, to generate the NOP56-RAN reporter plasmid, the NOP56-intron1 region, lacking an ATG start codon, was PCR-amplified from pUAST-attB-NOP56-GGCCTG-71 repeats using the primers HI_240 (5′-GAGGAGTGGTTGCGGGGCGCGGGCGACGCGAC-3′) and HI_090 (5′-TGCCGGAACCCGTTCCCAGGGCAGG-3′) with Prime STAR Max DNA Polymerase (Takara). The PCR conditions were 35 cycles of 10 s at 95 °C, 8 s at 58 °C, and 10 s at 72 °C, followed by 5 min at 72 °C. The PCR product was subcloned into the pcDNA5/FRT-T7-3FT vector (27). The size and integrity of the amplified repeat by PCR were verified by gel electrophoresis and sequencing. Various lengths of NOP56 GGCCTG repeats were randomly generated by PCR. To construct the NOP56-Nluc+3 × HA reporter plasmids (for *in vitro* translation), the Nluc+3 × HA region was amplified from the Nluc+3 × HA sequence (27) and inserted downstream of the pcDNA5_FRT-T7-NOP56 vector. Initiation codon-mutated reporters (Fig. 3*A*) were generated using PCR and Gibson assembly. For the human-transfected NOP56 reporter plasmids, Nluc-3 × FLAG was amplified and inserted downstream of the pcDNA-FRT-T7-NOP56 vector.

### *In vitro* transcription

Reporter mRNAs were synthesized as previously described (57). Reporter plasmids were linearized with XbaI (Takara), and the digested plasmids were purified using the Wizard SV Gel and PCR Purification Kit (Promega). 5′ m^7^G-capped mRNAs were synthesized using mMESSAGE mMACHINE T7 Transcription Kit (Thermo Fisher) and subsequently polyadenylated with *E*. *coli* poly(A) polymerase (NEB). 5′ A-capped mRNAs were synthesized using HiScribe T7 High Yield RNA Synthesis Kit (NEB) and G (5′) ppp (5′) A RNA Cap structure Analog (NEB). The mRNAs were purified by lithium chloride precipitation, and their size and integrity were evaluated through denaturing RNA gel electrophoresis.

### *In vitro* translation in RRL

mRNAs were translated *in vitro* using the Flexi Rabbit Reticulocyte Lysate system (Promega) as previously described (57). Translation reactions were conducted with 3 nM final mRNA and contained 30% RRL, 10 μM amino acid mix minus methionine, 10 μM amino acid mix minus leucine, 0.5 mM MgOAc, 100 mM KCl and 0.8 U/μl Murine RNase inhibitor (NEB). For 5′ cap dependency experiments, uncapped mRNA was used under the same conditions. Translation efficiency was evaluated using luminescence assay and Western blotting as previously described (27). For the luminescence assay, 2 μl of the sample was diluted in Glo lysis buffer (Promega) and mixed with NanoGlo substrate (diluted 1:50 in NanoGlo buffer) at a 1:1 ratio. After 3 min incubation in the dark with shaking, luminescence was measured using a Varioskan LUX Multimode Microplate Reader (Thermo Fisher Scientific). For Western blotting, 9 μl of each sample was mixed with 30 μl of 4 × SDS sample buffer (240 mM Tris-HCl pH 6.8, 40% (v/v) glycerol, 0.01% (w/v) bromophenol blue, 7% (w/v) SDS, 10% (v/v) of 2-mercaptoethanol) and heated at 70 °C for 15 min. Next, 15 μl of the samples were loaded onto a 13% SDS-polyacrylamide gel for electrophoresis. After separation, proteins were transferred onto PVDF membranes and blocked with 2% (w/v) skim milk in TBS-T and incubated for 1 h with primary antibodies listed in Table S3. Membranes were washed three times with TBS-T for 5 min each and incubated with HRP-conjugated anti-mouse IgG (Sigma-Aldrich). Chemiluminescence signals were detected using an LAS4000 system (Fuji Film) with Immobilon Western Chemiluminescent HRP reagent (Millipore).

### *In vitro* translation in HeLa lysate

*In vitro* translation using HeLa lysate driven from HeLa cells was performed as previously described (27, 58). Briefly, reactions were conducted with mRNAs at a final concentration of 10 nM and incubated at 32 °C for 2 h. In the m^7^GpppG competition assay, m^7^GpppG was added at a final concentration of 250 μM. Samples were incubated at 32 °C for 2 h and then placed on ice to terminate the reaction. The samples were analyzed using the same methods as those applied for *in vitro* translation in RRL.

### Cell culture and transfections

HeLa cells were cultured in Dulbecco’s Modified Eagle’s medium (DMEM; Nacalai Tesque) supplemented with 2 mM L-glutamine, 100 U/ml penicillin, 0.1 mg/ml streptomycin (Sigma-Aldrich), and 10% (v/v) fetal bovine serum (FBS; Thermo Fisher Scientific). For NOP56-RAN luminescence assays, HeLa cells were seeded in 96-well plates at a density of 2 × 10^4^ cells per well. After 24 h, cells were transfected with 50 ng Fluc control plasmid, 50 ng NOP56-RAN Nluc reporter plasmid, and 0.3 μl FuGENE HD (Promega) in 20 μl opti-MEM (Invitrogen) per well. After 24 h, cells were lysed in 50 μl Glo lysis buffer (Promega). For luminescence assays, 30 μl of lysate was incubated with 30 μl ONE Glo EX Fluc substrate in a 1:1 ratio for 3 min in the dark with shaking. Fluc luminescence was measured using a Varioskan LUX Mulimode Microplate Reader (Thermo Fisher Scientific). Following Fluc measurement, NanoGlo substrate diluted 1:100 in NanoGlo buffer (Promega) was added to the same plate, and Nluc activity was measured after shaking. For stress induction, HeLa cells were transfected with plasmids for 19 h, followed by treatment with 1 μM thapsigargin (Thermo Fisher Scientific, T7458) for 5 h. For Western blotting of RAN reporters, HeLa cells were seeded in 12-well plates at a density of 1 × 10^5^ cells per well. After 24 h, cells were transfected with 500 ng NOP56-RAN reporter plasmid using 1.4 μl FuGENE HD (Promega) and 50 μl OptiMEM (Invitrogen) per well. After 24 h, cells were harvested and lysed in 100 μl RIPA buffer (50 mM Tris-HCl pH 6.8, 150 mM NaCl, 0.1% (w/v) SDC, 0.1% (w/v) SDS, 1.0% (w/v) NP-40). Lysates (70 μl) were mixed with 30 μl 4 × SDS sample buffer and heated at 70 °C for 15 min. Samples were analyzed by Western blotting using the same procedure as for *in vitro* translation. For eIF2α and p-eIF2α detection, after separation, proteins were transferred onto PVDF membranes and blocked with 5% (w/v) bovine serum albumin (BSA) (Sigma-Aldrich) in TBS-T and incubated for 1 h with primary antibodies listed in Table S3. Membranes were washed three times with TBS-T for 5 min each and incubated with HRP-conjugated anti-Rabbit IgG (Sigma-Aldrich). Each experiment was performed in triplicate.

### Immunoprecipitation

100 μl *in vitro* translation reactions were performed using 10 nM mRNA in the RRL (Promega) at 30 °C for 90 min, followed by termination on ice. The samples were cleared by centrifugation at 20,000×*g* for 10 min at 4 °C, and the supernatants were transferred into new tubes containing 400 μl TBS (total volume 500 μl). Each sample was incubated with 10 μl pre-washed HA agarose beads (Pierce Anti-HA Agarose, 26,181) and rotated at 4 °C for 1 h. FLAG beads and HA beads were then washed three times with 500 μl TBS. For LC-MS/MS analysis, beads were resuspended in 50 μl Phase Transfer Surfactant (PTS) buffer (12 mM Sodium Deoxycholate (SDC), 12 mM Sodium *N*-lauroyl sarcosinate (SLS), 100 mM Tris-HCl, pH 9.0) and boiled at 95 °C for 5 min. The beads were cleared by column purification, and the flow-through was collected into a new tube. These samples were used for further analysis.

### Preparation for LC-MS/MS analysis

After immunoprecipitation of the RRL samples using HA agarose beads and eluted with PTS buffer, LC-MS/MS preparation was performed as previously described (59). First, the samples were reduced by treatment with 10 mM dithiothreitol (DTT) at room temperature for 30 min, then alkylated with 50 mM iodoacetamide in the dark at room temperature for 20 min. The protein mixture was then diluted 5-fold with 50 mM ammonium bicarbonate (ABC). For digestion of the denatured proteins into peptide fragments, 0.25 μg of Trypsin/Lys-C Mix (Promega, V5072) was added and incubated at room temperature for 3 h. An additional 0.5 μg of Trypsin/Lys-C Mix was added and incubated at 37 °C overnight. After digestion, an equal volume of ethyl acetate and 0.5% trifluoracetic acid (TFA) was added to the samples. The mixture was shaken vigorously for 2 min and centrifuged at 15,700×*g* for 2 min. The upper ethyl acetate layer was discarded, and the solvent was removed using a centrifugal evaporator. The residual pellet was redissolved in 80 μl of 0.1% TFA and 2% acetonitrile and desalted as follows: the solution was applied to a GL-Tip SDB (GL Sciences), equilibrated with 0.1% TFA and 2% acetonitrile, washed with 0.1% TFA and 2% acetonitrile, and eluted with 0.1% TFA and 80% acetonitrile. The solvent was removed by centrifugal evaporator, and the residual peptides were redissolved in 20 μl of 0.1% TFA and 2% acetonitrile. The solution was centrifuged at 20,000×*g* for 5 min, and 18 μl of the supernatant was collected into a new tube for LC-MS/MS analysis.

### LC-MS/MS

LC-MS/MS measurements were performed using an Easy-nLC1000 nanoflow liquid chromatography system and a Q-Exactive tandem mass spectrometer equipped with a nano-ESI ion source (Thermo Fisher Scientific). The trap column was a 2 cm × 75 μm capillary column packed with 3 μm C18-silica particles (Nikkyo Technos, Japan). The flow rate was set to 300 nl/min. Separation was conducted using a 10 to 40% linear acetonitrile gradient over 30 min in the presence of 0.1% formic acid. MS/MS data were acquired in data-dependent acquisition (DDA) mode, controlled by the Xcalibur 4.0 program (Thermo Fisher Scientific). The DDA settings were as follows: the resolution was 70,000 for the full MS scan and 17,500 for the MS2 scan; the AGC target was 3.0 × 10^6^ for the full MS scan and 5.0 × 10^5^ for the MS2 scan; the maximum IT was 60 ms for both the full MS and MS2 scans; the full MS scan range was *m/z* 310 to 1500, and the top 10 signals in each full MS scan were selected for the MS2 scan. DDA measurements were performed twice for each sample as technical replicates.

### Protein identification

The MS/MS data were analyzed using the Proteome Discoverer 2.4 software bundled with the Sequest HT search engine (Thermo Fisher Scientific) to obtain the MS/MS spectra and peptide search parameters. MS2 spectra were searched against all *Oryctolagus cuniculus* ORF sequences obtained from the UniProt database (https://www.uniprot.org/taxonomy/9986, downloaded on 20/3/2023) and predicted N-terminal candidate sequences listed in Table S4. The search parameters were as follows: MS1 and MS2 tolerance were set to 10 ppm and 0.2 Da, respectively; carbamidomethylation of cysteines (57.02146 Da) was considered a static modification, while acetylation of the protein N-terminus and oxidation of methionine (15.9949 Da) were dynamic modifications. The Percolator algorithm was used to determine the false discovery rate (FDR), and proteins/peptides with ≦ 1% FDR were retained for further analysis. MS/MS spectra assigned to the peptides of interest were manually examined (Table S4).

### Quantification and statistical analysis

All statistical analyses were performed using custom R code. The presented quantitative data represent the mean ± SD from a minimum of three or six independent experiments. For comparison of NLuc reporter luciferase activity, one-way ANOVA and Student’s *t* test were performed to confirm statistical difference between control and experimental groups.

## Data availability

Raw data files are available in the Mendeley Data repository (https://doi.org/10.17632/kc64gxd8r3.1).

## Supporting information

This article contains supporting information.

## Conflict of interest

The authors declare that they have no conflict of interests with the contents of this article.

## Supporting information

Supplemenatry Materials
