# Supplementary material for: Dissecting the mechanism of NOP56 GGCCUG repeat-associated non-AUG translation using cell-free translation systems
Source: J Biol Chem. 2025 Feb 25;301(4):108360. doi: 10.1016/j.jbc.2025.108360 (PMC11979933; doi:10.1016/j.jbc.2025.108360)
Supplement: Supplemenatry Materials [file mmc1.pdf]

Supporting Information

Dissecting the mechanism of NOP56 GGCCUG repeat-associated non-AUG translation using cell-free translation systems

Mayuka Hasumi, Hayato Ito, Kodai Machida, Tatsuya Niwa, Tomoya Taminato, Yoshitaka Nagai, Hiroaki Imataka, Hideki Taguchi*

*Correspondence author: Hideki Taguchi
Email: taguchi@bio.titech.ac.jp

This PDF file includes:

Figures S1 to S9
Tables S1- S4

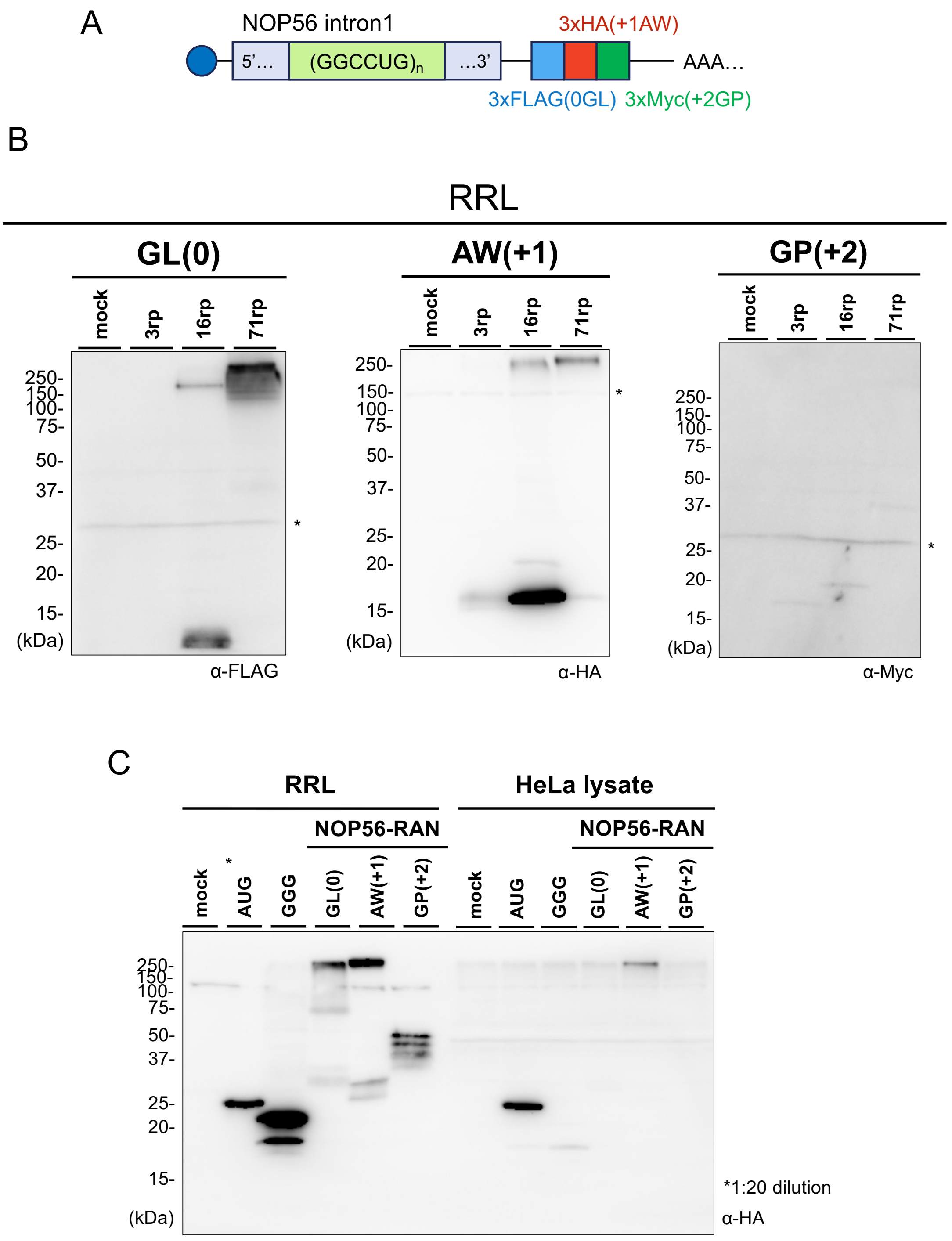

Supplementary Figure S1. NOP56-RAN translation in cell-free translation systems using nano-luciferase reporter.

(A) Schematic of the NOP56-3×frame-tag (FT) reporter. The FLAG, HA, and Myc tags were inserted in-frame with GL (0), AW (+1), and GP (+2) frames, respectively. (B) Western blot of the NOP56-3×FT reporter expressed in RRL. Dipeptide repeat (DPR)

products from all frames were detected. Predicted molecular weights of the products: GL (0): 7.2 kDa (3 repeats, rp), 9.4 kDa (16 rp), and 19 kDa (71 rp); AW (+1): 9.2 kDa (3 rp), 13 kDa (16 rp), and 27 kDa (71 rp); GP (+2): 12 kDa (3 rp), 14 kDa (16 rp), and 23 kDa (71 rp). Translation of GL (0) and AW (+1) frames produced proteins with molecular weights higher than predicted. (C) Western blot of the translation products from the NOP56-Nluc reporters expressed in RRL (reaction time: 90 min) and HeLa lysate (reaction time: 2 h).

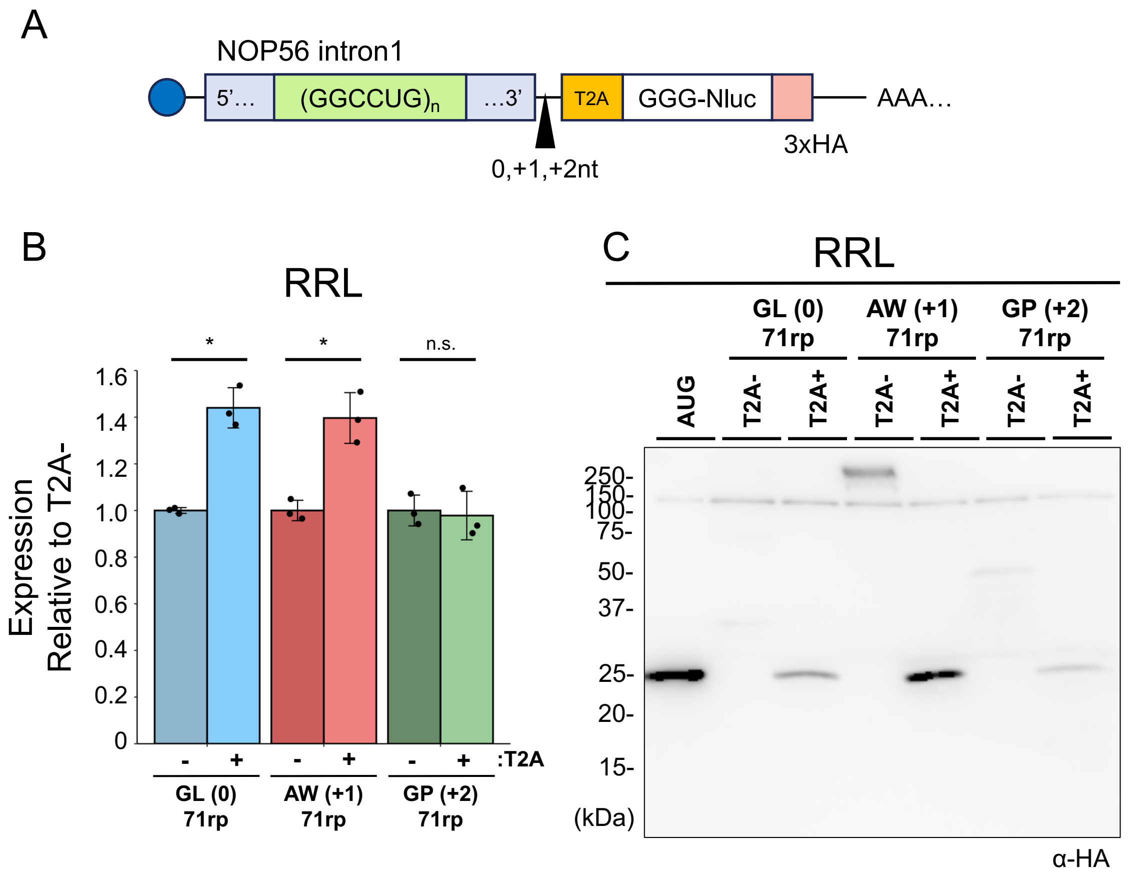

Supplementary Figure S2. NOP56-RAN translation in cell-free translation systems using nano-luciferase reporter.

(A) Schematic of the NOP56-T2A reporter. (T2A is the self-cleaving peptide from *Thosea asigna* virus 2A) (B) Expression levels of NOP56 reporters with T2A, normalized to the T2A- in RRL. Error bars represent ±SD from three independent experiments. n.s. = not significant; * $p < 0.05$, two tailed Student's t-test.

(C) Western blot of the NOP56-T2A reporter expressed in RRL. Dipeptide repeat (DPR) products from all frames were detected, confirming that the products were generated by the T2A cleavage. Predicted molecular weights of the products: *AUG* (AUG-Nluc); 25 kDa, *T2A-*: GL (0): 41 kDa, AW (+1): 46 kDa, and GP (+2): 39 kDa (same as Fig. 2B, C); *T2A+*; 25 kDa.

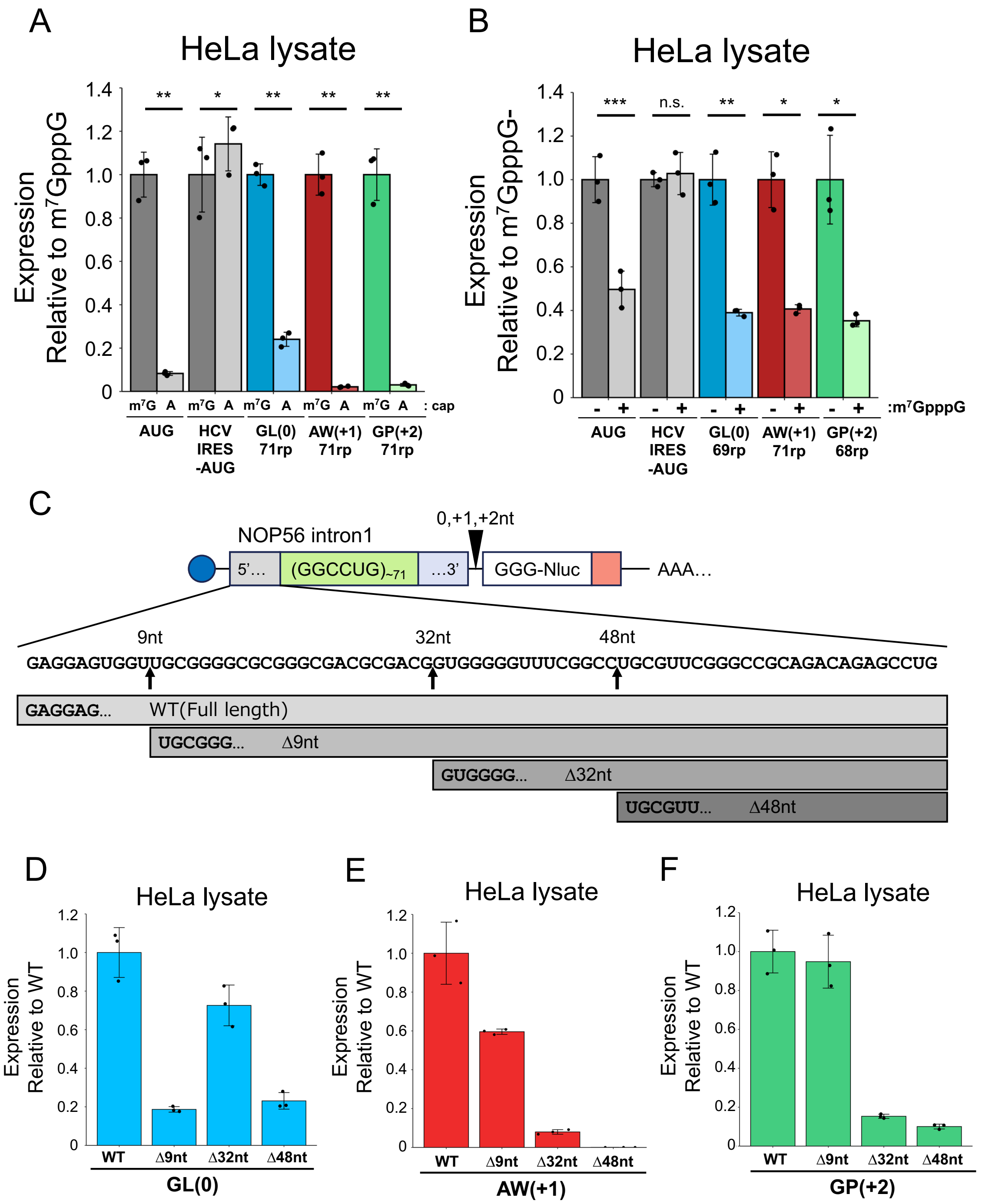

Supplementary Figure S3. Cap-dependency and truncation analysis of NOP56-RAN in HeLa lysate.

(A) m^{7}G; m^{7}GpppG capped, A-cap; ApppG capped. m^{7}G-cap dependency of translation in HeLa lysate for each frame using the NOP56-Nluc reporter. Canonical AUG-start translation (AUG+Nluc) and HCV-IRES (HCV IRES-AUG) were included as a control.

Error bars represent ±SD from three independent experiments. * p <0.05; ** p <0.01, two tailed Student's t-test. (B) Effect of the cap analog m^7GpppG on the translation of the NOP56-Nluc reporter. Luciferase reporters with AUG-start and IRES were also included as controls. Error bars represent ±SD from three independent experiments. n.s. = not significant; * $p < 0.05$; ** p <0.01; *** p <0.001, two tailed Student's t-test. (C) Schematic diagram of the reporters with truncated NOP56 upstream regions for approximate determination of initiation sites. The GGCCUG repeat length was ~71 repeats. (D-F) Expression levels of NOP56 reporters with upstream truncations, normalized to the intact reporter (WT) in HeLa lysate. Error bars represent ±SD from three independent experiments.

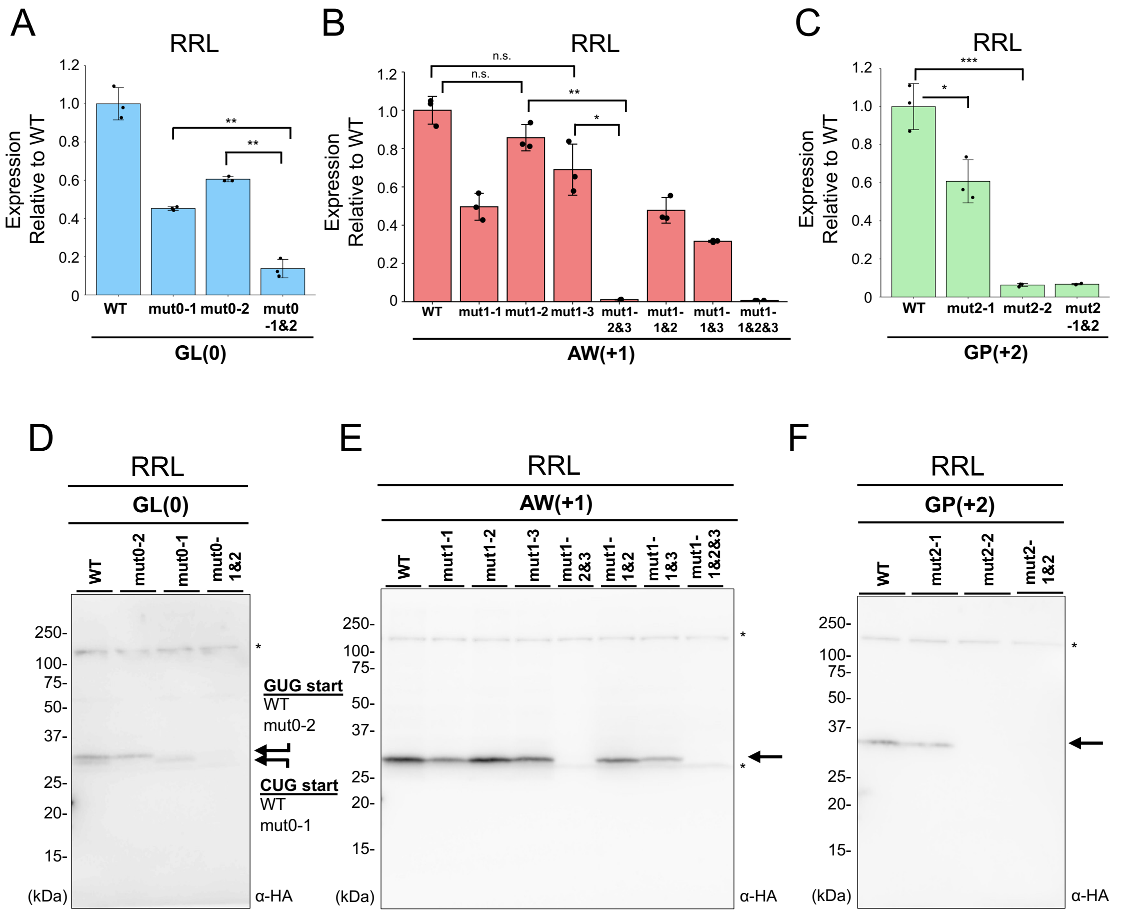

Supplementary Figure S4. Mutation analysis for identifying initiation sites in RRL translation.

(A-C) Expression levels of NOP56-RAN reporters with mutated start codons (Fig. 3A) relative to WT in RRL. The GGCCUG repeat lengths was 16 repeats. Error bars represent ±SD from three independent experiments. * p < 0.05; ** p < 0.01; *** p <0.001, two tailed Student's t-test. (A) GL (0), (B) AW (+1), and (C) GP (+2). (D-F) Western blot analysis of NOP56-RAN reporters with mutated start codons expressed in RRL. (D) GL (0), (E) AW (+1) and (F) GP (+2). Predicted molecular sizes of the products: GL (0): 31.6 kDa (GUG start) and 30.3 kDa (CUG start); AW (+1): 32.3 kDa (UUG start) and 31.7 kDa (ACG and GUG start); GP (+2): 30.4 kDa (ACG start).

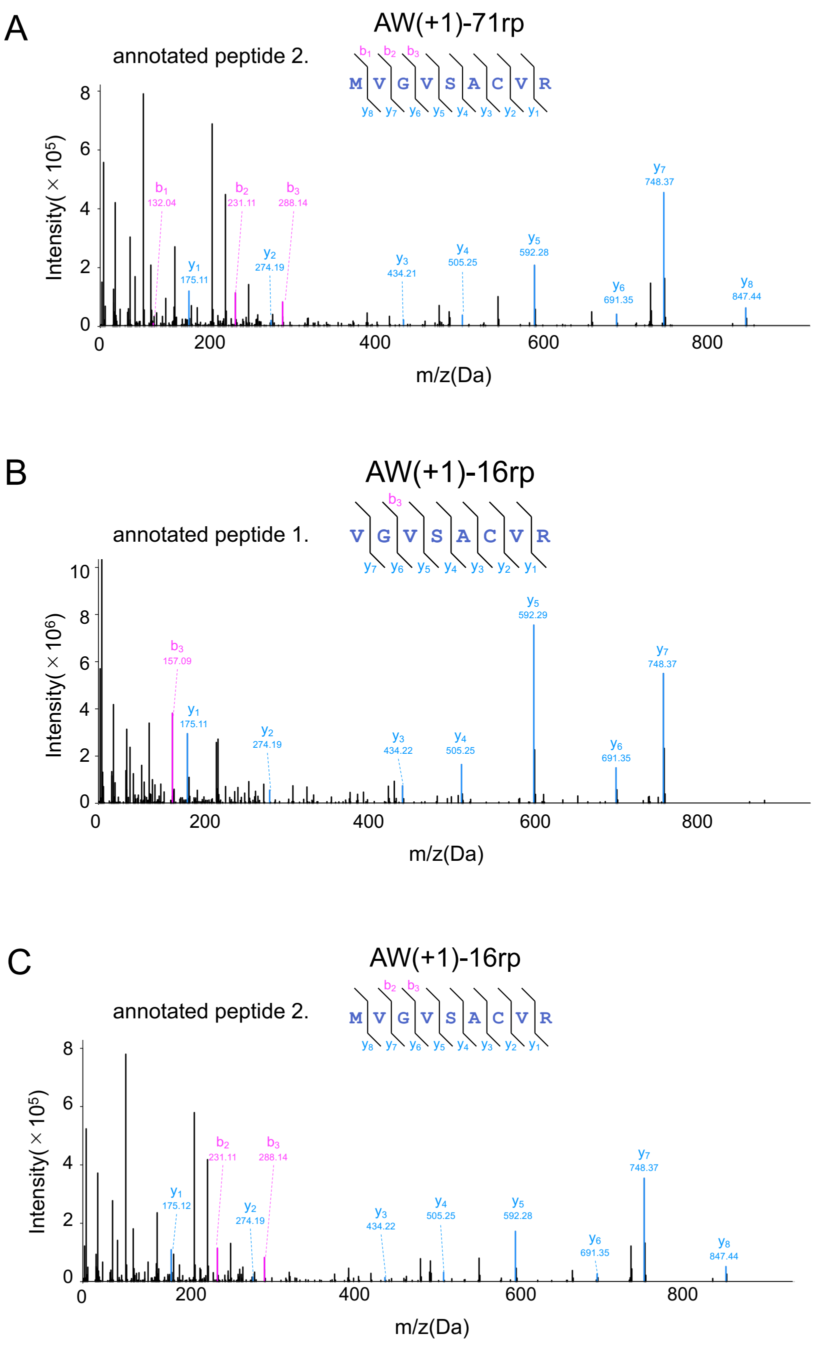

Supplementary Figure S5. LC-MS/MS analysis to identify initiation sites in the AW (+1) frame.

(A) LC-MS/MS spectrum of the N-terminal region of the HA-immunoprecipitated and trypsin-digested poly AW-71 repeat product expressed in RRL. Two peptides were identified: 1-VGVSACVR and 2-MVGVSACVR. The MS spectrum for peptide 2 is shown. (B, C) LC-MS/MS analysis of translation products with 16 repeats in the AW (+1)

frame. The analysis followed the same precedure as in (A) and Fig. 3E, detecting the same two peptides: 1-VGVSACVR (B) and 2-MVGVSACVR (C).

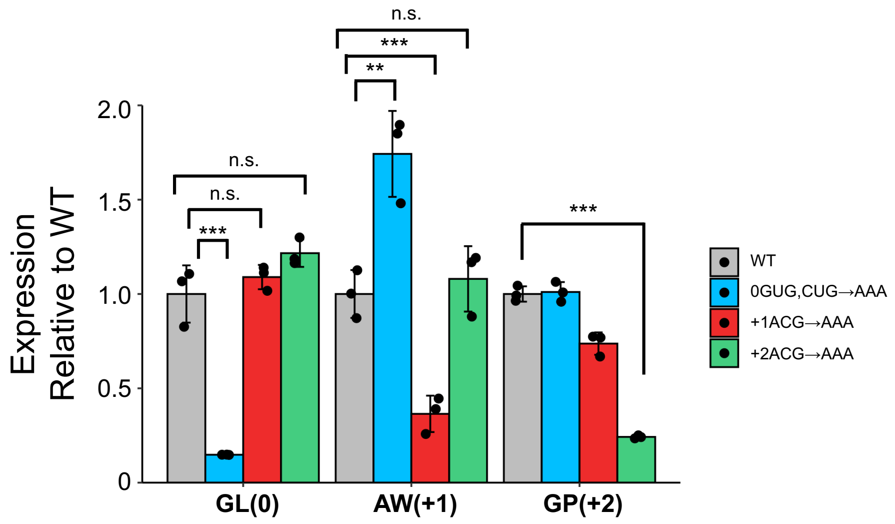

Supplementary Figure S6. Analysis of potential competition between frames.

(A) Analysis of each frame when the start codon in each frame was mutated to AAA. (GL (0): CUG, GUG; AW (+1): ACG; GP (+2): ACG). The GGCCUG repeat length was 16 repeats. Expression of the reporter is shown normalized relative to the wild-type for each frame. Error bars represent ±SD from three independent experiments. n.s. = not significant; ** $p < 0.01$; *** $p < 0.001$, two tailed Student's t-test.

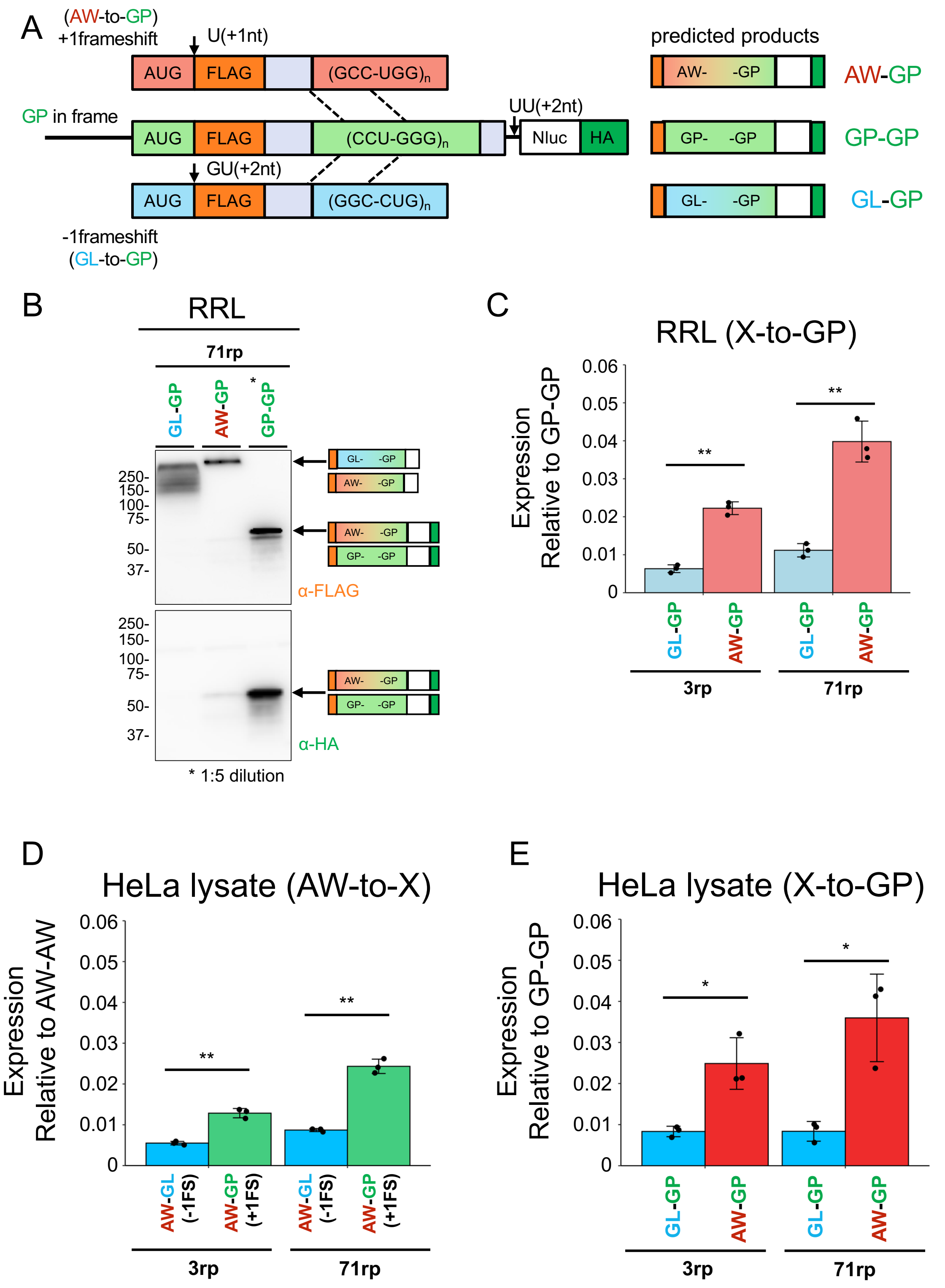

Supplementary Figure S7. X-to-GP frameshift analysis in NOP56-RAN.

(A) Schematic of reporters with an AUG driving expression through an N-terminal FLAG (orange) tag, followed by a C-terminal 3×HA (green) tag in GP, fused to the NOP56 intron

1 with 3 or 71 repeats (rp) (FLAG-NOP56-Nluc, HA(GP) reporter). (B) Anti-FLAG and anti-HA Western blot of FLAG-NOP56-Nluc, HA(GP) reporter mRNAs expressed in each frame in RRL. Predicted molecular sizes for anti-FLAG: GL-GP: smaller than 42 kDa; AW-GP smaller than 47 kDa; GP-GP in frame, 40 kDa. Predicted molecular sizes for anti-HA: GL-GP, not detected; AW-GP: 40-47 kDa; GP-GP in frame: 40 kDa. (C, E) Relative expression of FLAG-NOP56-Nluc, HA (GP) reporter normalized to GP-GP in-frame luciferase activity in (C) RRL and (E) HeLa lysate. Relative values are shown with luciferase activity for in-frame (GP-to-GP) set to 1. Error bars represent ±SD from three independent experiments. ** $p < 0.01$, two tailed Student's t-test. For the 71-repeat constructs, the luciferase activities of AW-GP and GL-GP relative to GP-GP were 3.6% and 0.8%, respectively. (D) Relative expression of FLAG (AW)-NOP56-Nluc, HA reporters normalized to AW in-frame AUG expression in HeLa lysate. Relative values are shown with luciferase activity for in-frame (AW-to-AW) set to 1. Error bars represent ±SD from three independent experiments. * $p < 0.05$, two tailed Student's t-test.

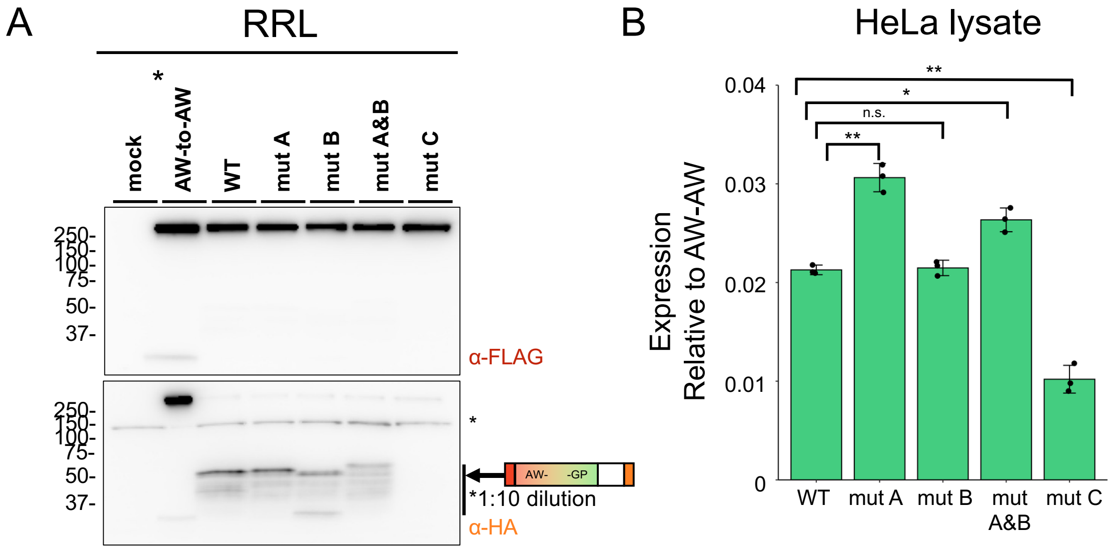

Supplementary Figure S8. Frameshift site analysis in NOP56-RAN.

(A) Anti-FLAG and anti-HA western blot of FLAG (AW)-NOP56-Nluc, H A(GP)-mutation reporters expressed in RRL. In the luciferase assay, frameshift products detected by HA-tag decreased when the GAAAG sequence was mutated (mut C). (B) Frameshift efficiency of AW-GP with mutation at the putative frameshift site. Relative expression of FLAG (AW)-NOP56-Nluc, HA reporters was normalized to AW-AW in-frame AUG expression expressed in HeLa lysate. Error bars represent ±SD from three independent experiments. n.s. = not significant; * p <0.05; ** p < 0.01, two tailed Student's t-test.

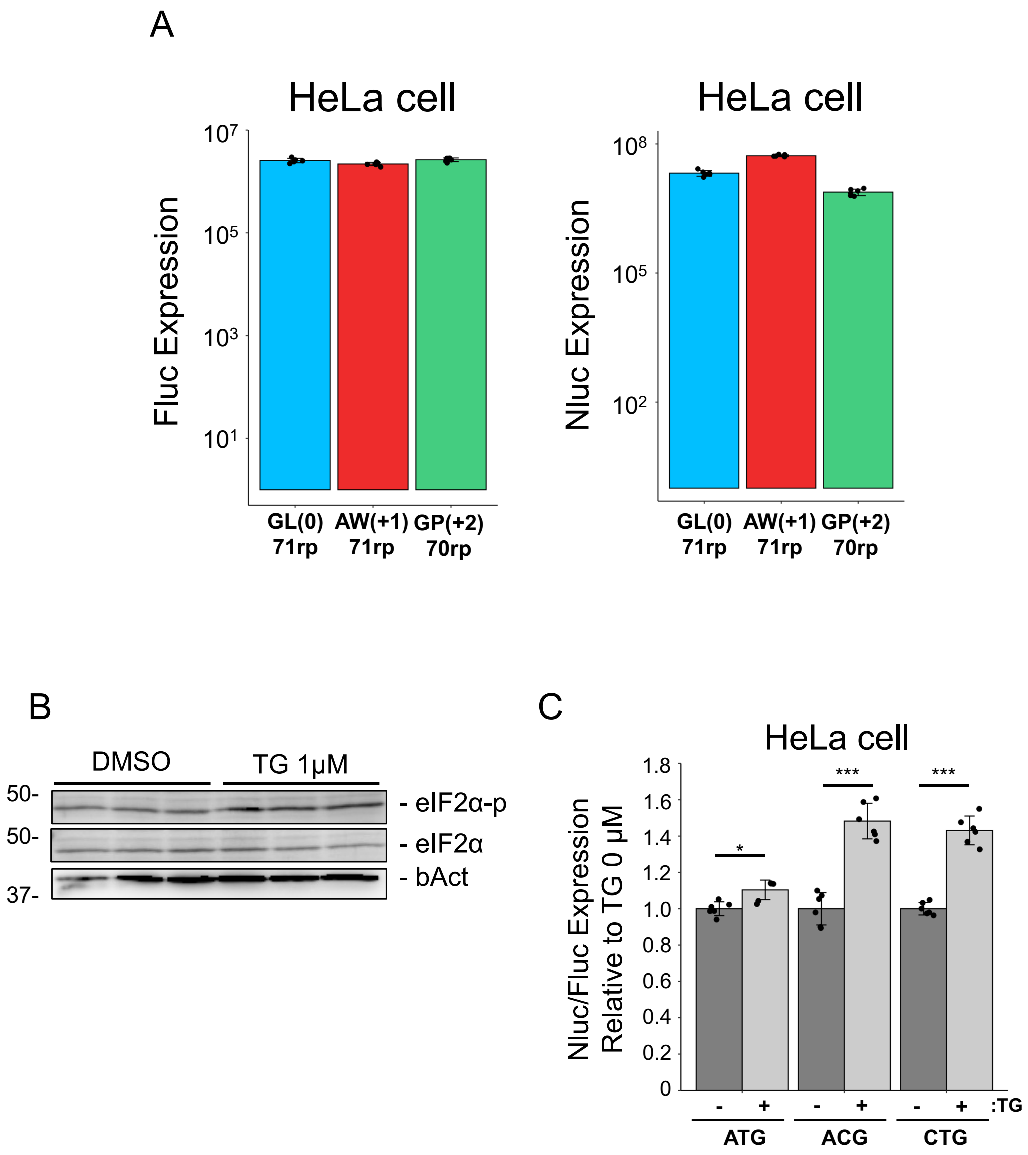

Supplementary Figure S9. NOP56-RAN in human cultured cells.

(A) Expression of ATG-Fluc and NOP56-Nluc reporters plasmids expressed in HeLa cells. ATG-Fluc control showed consistent expression across frames, while AW (+1) of Nluc exhibited the highest expression among the three frames, being 10-fold higher than GP (+2). Error bars represent ±SD from six independent experiments. (B) Western blotting analysis of HeLa cells after the treatment with 1μM thapsigargin (TG). eIF2α phosphorylation was detected by anti p-eIF2α, anti eIF2α and β-actin (bAct). bAct was used as a loading control. Three independent experiments are shown for each condition. (C) Relative Nluc/Fluc expression of NOP56-Nluc reporters normalized to TG- in HeLa cell. Error bars represent ±SD from six independent experiments. * $p < 0.05$; *** $p <$

0.001, two tailed Student's t-test. Translation of ATG-Nluc remained unchanged with or without TG treatment, whereas ACG/ CTG+Nluc expression increased by ~1.5 fold following TG treatment.
